# Editorial: Toxicity Mechanism and Clinical Features of PD-1/PD-L1 Inhibitors in Treatment of Cancer, Volume I

**DOI:** 10.3389/fphar.2022.898368

**Published:** 2022-05-05

**Authors:** Xiangyi Kong, Ryan J Sullivan, Caisheng Wu, Lin Zhang, Yu Jiang

**Affiliations:** ^1^ Department of Breast Surgical Oncology, National Cancer Center/National Clinical Research Center for Cancer/Cancer Hospital, Chinese Academy of Medical Sciences and Peking Union Medical College, Beijing, China; ^2^ Center for Melanoma, Massachusetts General Hospital Cancer Center, Harvard Medical School, Harvard University, Boston, MA, United States; ^3^ School of Pharmaceutical Sciences, Xiamen University, Xiamen, China; ^4^ School of Population Medicine and Public Health, Chinese Academy of Medical Sciences and Peking Union Medical College, Beijing, China; ^5^ Melbourne School of Population and Global Health, The University of Melbourne, Carlton, VIC, Australia; ^6^ Centre of Cancer Research, Victorian Comprehensive Cancer Centre Project, Melbourne, VIC, Australia

**Keywords:** PD-1/PD-L1 inhibitors, toxicity mechanism, clinical features, immunotherapy, cancer

The Research Topic titled “Toxicity Mechanism and Clinical features of PD-1/PD-L1 Inhibitors in Treatment of Cancer, Volume I” is focused on the clinical characteristics and the molecular or cellular biological mechanisms of PD-1/PD-L1 inhibitors in the treatment of various cancers. PD-1 and PD-L1 inhibitors are important targets in immunotherapy for cancer treatment. Anti-PD-1/PD-L1 antibodies have been developed to combat many cancers as a result of their success compared to conventional cancer treatment with monoclonal antibodies. There has been a total paradigm shift in the treatment of oncological malignancies as more anti-PD-1/PD-L1 agents have also been approved by the FDA (US) and the European Medicines Agency (EMA).

The increase in anti-PD-1/PD-L1 agents use has provoked the emergence of a new spectrum of toxicities. These toxicities depend on the individual patient and the specific type of anti-PD-1/PD-L1 agent used in the treatment. Numerous clinical trials and studies have investigated the treatment-related toxicities of PD-1 and PD-L1 inhibitors. Further research is required to fully understand the mechanism of these toxicities. In this Research Topic, leading experts from the field of cancer immunotherapy fully shared new findings and current concepts in the mechanisms of pharmacological toxicity induced by PD-1/PD-L1 inhibitors and explore the difference in the incidence of toxicities between patients treated with PD-1 or PD-L1 inhibitors and those treated without. In total, about 60 basic scientists and clinicians from several countries, including China, Japan, Australia, Czechia, etc., contributed 8 originals or review articles about current perspectives and findings in corresponding diseases. These articles cover a wide range of aspects in the clinical features and mechanisms of pharmacological toxicity induced by PD-1/PD-L1 inhibitors in all tissues as well as those in the event of treatment leading to discontinuation.

Among the 8 published articles on this topic, 5 were original researches and 3 were reviews. An observational study led by Kato et al. from Japan focused on the frequency of immune checkpoint inhibitor-induced vasculitides, one main type of vascular toxicity (Wang et al.). Using data from the Japanese Adverse Drug Event Report Database (from April 2004 to March 2020), they found the use of nivolumab showed a significant signal for vasculitides. Significant signals of polymyalgia rheumatica were found when the patients were treated with nivolumab, pembrolizumab, and ipilimumab. Further, the frequencies of nivolumab- and pembrolizumab-induced polymyalgia rheumatica were higher in patients aged ≥70 years and female patients, respectively. This study highlights that careful monitoring for polymyalgia rheumatica is required when the patients are aged >70 years and have been treated with nivolumab and when the patients are women and have been treated with pembrolizumab.

Similarly, through a large-scale pharmacovigilance analysis, Zhang et al. investigated the associations between pancreatic toxicities and PD-1/PD-L1 Inhibitors and characterized the main features of related pancreatic injury based on the Food and Drug Administration Adverse Event Reporting System (FAERS) database (Zhang et al.). The strongest signal associated with pancreatitis was reported for anti-PD-L1, whereas that with diabetes mellitus was reported for anti-PD-1. Compared with monotherapy, combination therapy showed stronger associations with both pancreatitis and diabetes mellitus, but lower fatality proportion.

The other 3 original studies explored the PD-1/PD-L1 Inhibitors-related clinical practices in various certain cancer types. Li et al. aimed to adapt the Gustave Roussy Immune Score (GRIm-Score) in hepatocellular carcinoma (HCC) patients who received ICIs therapies and thus improved the predictive ability (Li et al.). In the validation group of 80 patients, the patients presenting a high score showed an inferior overall survival (OS) (*p* = 0.024). HCC-GRIm-Score had the highest area under curve of 0.719 compared to the original GRIm-Score and Barcelona clinic liver cancer (BCLC) staging system. Chen et al. investigated the correlations between controlling nutritional status [CONUT, based on total lymphocyte count (TL), total cholesterol level (T-CHOL), and serum albumin (ALB)] and prognosis in gastric cancer patients receiving PD-1/PD-L1 Inhibitors treatment (Chen et al.). With the PD-1/PD-L1 positive expression, the patients with high CONUT score had shorter progression-free survival (PFS) and OS than those with low CONUT score. Furthermore, the patients with high CA724 value were associated with shorter PFS and OS. Anemia was the main toxicity in gastric cancer patients receiving PD-1/PD-L1 Inhibitors.

By a retrospective study, Sun et al. explored the efficacy and adverse events in patients with advanced Urothelial Carcinoma (UC) treated with PD-1 inhibitors (Sun et al.). They reviewed 118 patients who met the inclusion criteria. A sub-analysis according to the treatment method (PD-1 inhibitor vs PD-1 inhibitor plus chemotherapy) was performed. The incidence of toxicities at Grade 1–2 was not different between the groups (85 vs 94%), but combination therapy significantly increased toxicities at Grade 3–4 (32 vs 89%). In conclusion, PD-1 inhibitors show promising tolerance and efficacy in advanced UC. PD-1 inhibitors combined with chemotherapy offered better disease control but had more serious toxicities. The clinical use of combination therapy warrants caution.

Also focusing on UC patients, Wang et al. conducted an updated review of toxicities in metastatic UC (mUC) patients treated with PD-1/PD-L1 Inhibitors and cytotoxic T-lymphocyte antigen 4 (CTLA-4) inhibitors based on current clinical trial data, and discussed therapeutic strategies used to monitor and deal with such toxic reactions (Wang et al.). The toxicities in the general system, integumentary system, gastrointestinal system, hepatic system, endocrine system, and respiratory system were summarized in detail. On the whole, PD-1/PD-L1 Inhibitors have demonstrated good safety in mUC treatment, with manageable. However, doctors should endeavor to acquire more immunological knowledge and make immediate diagnoses based on the manifestations of toxicities.

Among various toxicity types, myocarditis induced by anti PD-1/PD-L1 agents is uncommon, but potentially fatal toxicity. A timely and informative mini-review article by Dong and Kong et al. summarized the incidence, characteristics, diagnosis and treatments, as well as illustrated the potential pathogenesis from the perspectives of T lymphocytes infiltration, disturbance of regulatory T cells, cytokines, macrophages mediated inflammatory response, and synergistic effect of PD-1/PD-L1 Inhibitors and CTLA-4 inhibitors (Dong et al.).

Another key review article was provided by Hradska et al. from Czechia (Hradska et al.). Their team reviewed all prospective blood cancer clinical trials investigating PD-1/PD-L1 Inhibitors (both monotherapy and combination therapy) with available toxicity data to determine the incidence of related toxicities in this specific setting and to offer a brief insight into their management, as the use of PD-1/PD-L1 Inhibitors was not so frequent in hematological malignancies. Generally, the incidence and distribution of these toxicities were approximately similar between solid tumor oncology and hemato-oncology. However, the exact rates might differ due to smaller amounts of treated patients and the fact that most of the safety data came from phase 1 and 2 clinical trials.

Finally, in line with its main goal, this Research Topic contributes to an “in-depth” knowledge of the toxicity mechanism and clinical features of PD-1/PD-L1 inhibitors in the treatment of cancer. Although current findings are only the tip of the iceberg, we strongly believe that this collection of articles exploring the complex world of toxicities related to PD-1/PD-L1 inhibitors, as well as their potential control methods and prevention strategies, will inspire many researchers and clinicians worldwide to continue working in or enter the exciting field of cancer immunotherapy.

